# Biofilm producing indigenous bacteria isolated from municipal sludge and their nutrient removal ability in moving bed biofilm reactor from the wastewater

**DOI:** 10.1016/j.sjbs.2021.06.084

**Published:** 2021-07-01

**Authors:** Khaloud Mohammed Alarjani, Abeer M. Almutairi, Subhanandharaj Russalamma Flanet Raj, Jayarajapazham Rajaselvam, Soon Woong Chang, Balasubramani Ravindran

**Affiliations:** a Department of Botany and Microbiology, College of Science, King Saud University, Riyadh 11451, Saudi Arabia; b Science Department, College of Basic Education, Public Authority for Applied Education and Training, (PAAET), Alardyia, Kuwait; c Department of Zoology, Nesamony Memorial Christian College, Marthandam, Kanyakumari, Tamil Nadu 629 165, India; d Bioprocess Engineering Division, Smykon Biotech Pvt LtD, Nagercoil, Kanyakumari, Tamil Nadu 629201, India; e Department of Environmental Energy and Engineering, Kyonggi University, Youngtong-Gu, Suwon, Gyeonggi-Do 16227, South Korea

**Keywords:** Biofilm, Nutrient, Phosphate, Wastewater, Treatment, Biofilm reactor

## Abstract

In the present study, improved moving bed biofilm reactor (MBBR) was applied to enhance the nutrient removal ability of the municipal wastewater. A total of 18 indigenous bacterial isolates were screened from the sewage sludge sample and nitrate reductase, nitrite reductase and hydroxylamine oxidase was analyzed. The strains *Pseudomonas aeruginosa* NU1 and Acinetobacter calcoaceticus K12 produced 0.87 ± 0.05 U/mg and 0.52 ± 0.12 U/mg hydroxylamine oxidase, 1.023 ± 0.062 U/mg and 1.29 ± 0.07 U/mg nitrite reductase, and 0.789 ± 0.031 U/mg and 1.07 ± 0.13 U/mg nitrate reductase. Nitrogen and phosphate removal improved by the addition of nutrient sources and achieved > 80% removal rate. pH and temperature of the medium also affected nutrient removal and improved removal was achieved at optimum level (p < 0.05). MBBR was designed with R1 (aerobic), R2 and R3 (anoxic) reactors. MBBR reactors removed acceptable level phosphorus removal properties up to 7.2 ± 3.8%, 42.4 ± 4.6%, and 84.2 ± 13.1% in the R1, R2, R3 and R4 reactors, respectively. Denitrification rate showed linear relationship at increasing concentrations nitrogen content in the reactor and denitrification rate was 1.43 g NO_2_-N /m^2^/day at 1.5 g NO_2_-N /m^2^/day. Dehydrogenase activity was assayed in all reactors and maximum amount was detected in the aerobic biofilm reactor. Based on the present findings, MBBRs and the selected bacterial strains are useful for the degradation domestic wastewater with minimum working area.

## 1 Introduction

Municipal wastewater contains phosphate, nitrate and other contaminants which mainly discharged directly to the environment without any treatment severely affects ecosystem and the health of human population (Saha et al., 2018). The composition of wastewater is highly complex and it contains mixture of highly complex molecules, which affect biodegradation process (Heidrich et al., 2011). Wastewater recycling process shows various benefits to the environment. It can be used either as a supplementary water for irrigation in the case of liquid wastewater or as a nutrient source for agricultural use. When this wastewater is used as irrigation process it would specifically meet nutrient demands and crop-water requirements (Tesfamariam et al., 2015). In agricultural sector, phosphorus (P) and nitrogen (N) are very important elements that are widely used (Prabhu and Mutnuri, 2019). These nutrients contributed to eutrophication and involved in the reduction of dissolved oxygen (DO) in water (Khazaei et al., 2016). These nutrient sources playing significant roles in agricultural sectors and are very important for plant growth and yield (Yang et al., 2011). Various biological, chemical and physical methods have been proposed for the removal of nutrients from the contaminated wastewater (Cao, 2016).

The moving bed biofilm reactor (MBBR) is widely used method for the removal of nutrients from the wastewater (Lariyah et al., 2016). This MBBR bioreactor has several advantages because of application of suspended solid materials that supports attached and biofilm producing bacteria. MBBR system effectively reduces the requirement of large wastewater treatment plants, and within very short hydraulic retention time increase cell residence (Casas et al., 2015). Wastewater treatment method using MBBR technology is useful to treat wastewater remediated by physical and biological methods for producing effluents which is useful for irrigation (Lin, 2018). Moreover, the phosphorus and nitrate concentration tend to specifically accumulate to a high concentration in MBBR (Sonwani et al., 2019). Biological methods based on the development of biofilm have been proved to offer effective removal of various organic substances and nitrogenous compounds from wastewater, minimizing problems associated with biomass recycling, usage for settling tanks and very large reactor size (McQuarrie and Boltz, 2011). The removal ability of nutrients varied based on the types of reactor, operation condition, hydraulic retention time and types of biomass used for operation. Development of biofilm in aerobic reactor and synthesis of various enzymes for the hydrolysis of organic matters from the wastewater is prime importance. In this study two biofilm forming bacteria were isolated from municipal sludge sample for the removal of nutrients from the wastewater in MBBR.

## 2 Materials and methods

### 2.1 Culture media

The basal medium applied for the screening and isolation of nitrifying bacteria consisted of the following composition (g/L): (NH_4_)_2_SO_4_ − 0.47; C_4_H_4_Na_2_O_4_·6H_2_O − 5.62 and trace element stock (50 mL). The initial pH of the culture was 7.5. The trace element stock solution was prepared using various elements. These include (g/L): MnSO_4_·4H_2_O – 0.5; FeSO_4_·7H_2_O – 0.5; NaCl – 2.5; MgSO_4_·7H_2_O – 2.5; and K_2_HPO_4_ – 5. The denitrification medium (BM medium) used for this study contained (g/L) MgSO_4_·7H_2_O – 0.1; KH_2_PO_4_ – 1.5; Na_2_HPO_4_·7H_2_O – 7.9; C_4_H_4_Na_2_O_4_·6H_2_O – 5.62; KNO_3_ – 0.72 and mineral salt solution (2 mL). The mineral salt solution was prepared by adding (g/L) 50 g Na_2_.EDTA,1.57 g CuSO_4_·5H_2_O, 5.06 g MnCl_2_·4H_2_O, 5.5 g CaCl_2_, 2.2 g ZnSO_4_·7H_2_Oand 1.6 g CoCl_2_·6H_2_O.

### 2.2 Isolation of bacteria

Municipal soil sludge (1.0 g) sample was aseptically transferred to an autoclaved physiological saline (9 mL) in a 100 mL Erlenmeyer flask and kept on an orbital shaker at 100 rpm for 30 min and homogenous suspension was obtained. Serial dilutions were performed up to 10^−7^ dilution and the final diluted cultures were carefully spread onto BM agar medium. Then the plates were kept for 48–72 h and visible bacterial isolates were observed. The developed isolated single bacterial colony was picked and characterized for its nitrifying and denitrifying ability.

### 2.3 Enzyme assay

The isolated 18 morphologically distinct bacterial strains were cultured in BM medium without agar. Briefly, a loop full culture of predetermined bacterial strain was inoculated in to Erlenmeyer flask containing 50 mL BM broth. It was incubated at 32 ± 2 °C for 48 h and the culture was centrifuged. The harvested pellet was washed three times with phosphate buffered saline and cell-free extract was obtained after ultrasonic treatment. The reduction of nitrite, formation of nitrite from nitrate and reduction of NH_2_OH in the medium was used to determine nitrite reductase, nitrate reductase and hydroxylamine oxidase (Zhao et al., 2010a, Zhao et al., 2010b). Total protein content of the sample was evaluated as described previously (Lowry et al., 1951). Enzyme activity was expressed as U/mg protein (Wang et al., 2020). From these 18 bacterial strains only two bacteria were selected for characterization studies.

### 2.4 Characterization of bacterial strains

The strains with the highest nitrification and denitrifying activities were characterized. The morphological and biochemical characterization was performed as described previously. Then the total genomic DNA of the selected bacterial strains NU1 and KI2 were extracted using a genomic DNA extraction kit as described by the manufactures. The 16S rDNA genes of the selected bacterial strains (NU1 and KI2) were amplified.

### 2.5 Antibiotic-resistance properties of the bacterial strains

Antibiotic-resistance properties of the two selected strains were studied. The selected bacterial strains were grown on MHA medium and placed various commercial antibiotics. After 24 h, the resistance property was tested (Atif et al., 2020).

### 2.6 Artificial municipal wastewater and removal of nutrients

Synthetic wastewater was prepared by mixing the nutrients with tap water along with micro- and macro-nutrients. Two bacterial strains (*Pseudomonas aeruginosa* NU1 and Acinetobacter calcoaceticus K12) were selected because these bacterial strains were involved in activated sludge process for waste water. The removal nutrients (NH_4_^+^ - N, NO_3_^−^ - N, NO_2_^−^ - N and PO_4_^3−^) were determined by using synthetic municipal wastewater. It was prepared by mixing glucose (500 mg/L), NaHCO_3_ (550 mg/L), KH_2_PO_4_ (30 mg/L), K_2_HPO_4_ (80 mg/L) and NH_4_Cl (380 mg/L). The initial concentration of phosphorus and nitrogen were 20 mg/L and 100 mg/L, respectively. The COD level of the prepared artificial wastewater was 550 mg/L. Total alkalinity of the sample was 400 mg/L and total inorganic carbon content was 100 mg/L. The cultures were inoculated in to artificial municipal wastewater and incubated for 96 h and nutrient removal efficiency (%) was determined.

### 2.7 Physico-chemical parameters of municipal wastewater

Municipal wastewater was collected and the physico-chemical and nutrient factors were analyzed. The collected sample was filtered and used in MBBR. pH was analyzed *in situ* using a multi-parameter analyzer. The factors such as, BOD, COD, TSS, Al, Fe, Cd, N-NO_3_ and P-PO_4_ were determined.

### 2.8 Growth of bacteria in municipal wastewater

The growth of two selected bacterial strains (*P. aeruginosa* NU1and A. calcoaceticus K12) was monitored for six days. The isolated bacterial strains were inoculated in a sterilized municipal wastewater supplemented with 0.5% (w/v) glucose. It was incubated for 6 days at 37 °C. The culture was withdrawn for every 24 h and was cultured on nutrient agar medium. After 24 h, bacterial colonies were counted using an automated colony counter and colony forming unit (CFU) was calculated (Vijayaraghavan et al., 2019).

### 2.9 Moving bed biofilm reactor (MBBR)

The experimental set up contains four reactors namely, anaerobic reactor (R1), first anoxic reactor (R2), second anoxic reactor (R3) and aerobic reactor (R4) connected in sequence manner. Phosphorus removal was mainly initiated in anaerobic reactor R1. The reactor R2 was applied to reduce the impact of nitrate content in wastewater and this chamber naturally contains more COD. Anoxic recirculation (AR) was applied to enhance utilization of organic matters from the wastewater and the flow rate was improved than influent flow rate. The bioreactor R3 and R2 received water inflow from the aerobic reactor (R2) and involved in the removal of nitrate. The operating temperature of the MBBR was 30 ± 2 °C. The schematic representation of moving bed bioreactor is described in Fig. S1. Samples were collected from sampling mode from all reactors. The propeller speed of the reactor was 30 rpm. A rotameter was used to measure the air inflow and manually controlled using a valve. Aerobic reactor was fed with *P. aeruginosa* NU1and A. calcoaceticus K12 for 20 days for the development of bacterial biofilm before to start the experiment. These two bacterial strains have the ability to grow in wastewater based on previous analysis. To enrich the wastewater, glucose and ammonium nitrate were added. These two nutrients were suitable to improve nutrient removal from the wastewater by *P. aeruginosa* NU1 and A. calcoaceticus K12. The COD of the wastewater ranged between 700 and 1100 mg/L, and various concentrations of NH_4_-N ranged between 10 and 200 mg/L and PO_4_-P ranged between 2.5 and 30 mg/L. The DO content of the wastewater ranged between 2.0 and 6.0 mg/L depending on the wastewater inflow. Wastewater was pumped in to the reactors continuously with flow rate of 10 L/day. The predicted Hydraulic Retention Time in the anaerobic/anoxic and aerobic reactor was 6 and 18 h, respectively.

### 2.10 Dehydrogenase activity of the culture in the bioreactor

Dehydrogenase activity was assayed by standard method. The sample was collected from the all four bioreactor. About 5 mL sample was mixed with 2,3,5-triphenyl-tetrazolium chloride (TTC) solution at various concentrations (0.5–1.0% TTC). To the blank, Tris buffer (pH 7.2) was added. Then triphenylformazan was extracted with methanol and culture supernatant was transferred in to new vials (Alef, 1995).

### 2.11 Statistical analysis

The data were presented as mean ± standard deviation. One way ANOVA was used to test the significance difference using the statistical software, SPSS.

## 3 Results and discussion

### 3.1 Characterization of bacteria and enzymes

A total of eighteen bacteria were isolated from the sludge sample and determined for their potential to synthesize various enzymes. Two bacterial strains were used for this study. Hydroxylamine oxidase activity was maximum in strain NU1 and the enzyme activity was 0.74 U/mg protein. Maximum nitrite- and nitrate-reductase activities were determined in strain K12 (1.29 U/mg protein and 0.98 U/mg protein). The production of enzymes by the selected all eighteen bacterial strains were described in Table 1. Huang et al. (2013) reported the ability of nitrate and nitrite utilization by *Acinetobacter* sp. Y16 isolated from oligotrophic niche. This organism showed the ability to synthesize hydroxylamine oxidase and determined enzyme activity in the culture (0.03 U/mg protein). The production ability of enzyme was found to be higher than previous studies. Due to their potential properties, aerobic denitrifying - heterotrophic nitrifying bacteria have been used in the treatment of nitrogenous wastewater (Yao et al., 2013).

**Table 1:** Analysis of nitrate reductase, nitrite reductase and hydroxylamine oxidase of bacterial strains isolated from the sludge sample.

Stains	Enzyme activity (U/mg protein)		
	Hydroxylamine oxidase	Nitrite reductase	Nitrate reductase
D9	0.59 ± 0.02	0.57 ± 0.35	0.121 ± 0.03
D17	0.08 ± 0.0	0.052 ± 0.22	1.03 ± 0.05
D39	0.73 ± 0.03	0.093 ± 0.06	0.098 ± 0.02
K12	0.52 ± 0.12	1.29 ± 0.07	1.07 ± 0.13
K30	0.02 ± 0.0	0.97 ± 0.39	0.29 ± 0.06
K43	0.38 ± 0.12	1.06 ± 0.062	0.38 ± 0.05
LL08	0.83 ± 0.27	1.09 ± 0.001	0.53 ± 0.01
LL05	0.07 ± 0.2	0.008 ± 0.002	0.031 ± 0.03
LL92	0.64 ± 0.19	0.087 ± 0.011	0.092 ± 0.001
NU1	0.87 ± 0.05	1.023 ± 0.062	0.498 ± 0.031
NU17	0.003 ± 0.0	1.19 ± 0.031	0.939 ± 0.091
NU28	0.059 ± 0.04	1.01 ± 0.01	1.01 ± 0.02
OS29	0.76 ± 0.08	0.0089 ± 0.01	0.092 ± 0.051
OS32	0.53 ± 0.06	0.087 ± 0.03	0.08 ± 0.019
OS44	0.083 ± 0.03	0.035 ± 0.04	0.52 ± 0.13
RS92	0.65 ± 0.07	0.018 ± 0.001	0.98 ± 0.021
RS103	0.048 ± 0.02	0.172 ± 0.07	0.082 ± 0.002
RS108	0.69 ± 0.12	0.287 ± 0.021	0.049 ± 0.006

### 3.2 Isolation and characterization of bacteria

The isolated bacteria were analyzed for their potential to mediate heterotrophic nitrification and aerobic denitrification process. Among these, two bacterial strains, NU1 and K12 showed maximum efficiency for ammonium and nitrate removal from the culture medium. These selected strains (NU1 and K12) were characterized as *P. aeruginosa* NU1 and A. calcoaceticus K12 based on morphological, biochemical and 16S rDNA sequencing. The morphological and biochemical characters of these strains were described in Table 2. The members of the genus *Pseudomonas* and *Acinetobacter* have the ability to remove nitrogen and other nutrients from the environment. In the natural environment, *Pseudomonas* is one of the most abundant species and played potent role in denitrification and nitrification process under extreme temperature (Yao et al., 2013). Yang et al. (2015) characterized twenty bacterial strains isolated from the enriched medium and were analyzed for their ability to involve in aerobic denitrification and heterotrophic nitrification process. Microbial population varied in wastewater treatment plants (WWTPs) in genus level between geographical locations. The genus, *Arcobacter* and *Acinetobacter* were dominant genera in WWTPs (Marti et al., 2013). The total microbial community in the WWTPs reflected industrial waste and human microbiome (Fisher et al., 2015).

**Table 2:** Physiological, morphological and biochemical properties of *P. aeruginosa* NU1 and A. calcoaceticus K12.

Experiments	*P. aeruginosa* NU1	*A. calcoaceticus* K12
*Biochemical characters*		
Gram's staining	Negative	Negative
Capsule	Non-capsulated	Non-capsulated
Citrate	Positive	
Gas-production	Negative	Negative
Hemolysis	Beta Hemolytic	Non-Hemolytic
Indole	Negative	Negative
Motility	Motile	Non-motile
Oxidase	Positive	Negative
Shape	Rod	Rod
Spore	Non-spore forming	Non-spore forming
Triple sugar ion test	Alkali	Alkali
Urease	Negative	Negative
*Carbohydrate fermentation*		
Fructose	Positive	Positive
Glucose	Positive	Positive
Starch	Negative	Negative
Sucrose	Negative	Negative
Maltose	Negative	Positive
Trehalose	Negative	Negative
Ribose	Positive	Negative
Xylose	Negative	Positive
*Antibiotic sensitivity*		
Ampicillin	Resistant	Resistant
Ciprofloxacin	Resistant	Resistant
Amikacin	Resistant	Resistant
Chloramphenicol	Resistant	Resistant
Meropenem	Resistant	Sensitive

### 3.3 Antibiotic-resistance properties of the bacterial strains

Antibiotic-resistance properties of the two selected strains were studied. The results of the antibiotic-resistant bacteria in the wastewater revealed that drug resistant bacteria could be widely distributed in the wastewater environment. *P. aeruginosa* NU1 and *A. calcoaceticus* K12 were resistant against ampicillin, amikacin, chloramphenicol and ciprofloxacin. Bacteria isolated from the effluent, including lactose-fermenting *Acinetobacter*, *Enterococcus* and Enterobacteriaceae showed high rates of resistant against rifampicin, tetracycline, chloramphenicol, cephalothin, and ampicillin (Huang et al., 2012, Wu et al., 2020). The selected strains showed antibiotic resistance against various drugs. Likewise, many functional microbes including, Aequorivita, Tissierella, Comamonas, Clostridiales bacterium*,* Comamonadaceae bacterium showed potent role in the removal of complex nitrogen and carbon source (Liu et al., 2020).

### 3.4 Effect of carbon and nitrogen sources on nutrient removal by bacterial strains

In *P. aeruginosa* NU1, at 4% glucose concentrations**,** 90.3 ± 1.1% NH_4_^+^ - N, 90.9 ± 2.2% NO_3_^−^ - N, 82.8 ± 1.9% NO_2_^−^ - N and 90.4 ± 2.2 PO_4_^3−^ were removed from the treated wastewater. At 0.1% ammonium nitrate concentration in the wastewater medium enhanced >85% nitrogen removal from the wastewater (Table 3a). In the case of A. calcoaceticus K12, 2% supplemented glucose removed 80.5 ± 5.5% NH_4_^+^ - N, 70.7 ± 5.1% NO_3_^−^ - N, 84.5 ± 5.9% NO_2_^−^ - N, and 87.4 ± 2.9% PO_4_^3−^ from the wastewater. When the glucose concentration increased from 2% to 5%, the nutrient removal rate decreased as 65.4 ± 4.7%, 60.7 ± 0.5%, 73.2 ± 1.4% and 70.4 ± 6.8% for NH_4_^+^ - N, NO_3_^−^ - N, NO_2_^−^ - N and PO_4_^3−^, respectively. Supplemented ammonium nitrate at 0.2% level significantly improved nutrient removal from the medium. Carbon and nitrogen sources serve as electron and energy sources for all heterotrophic bacteria. The removal of nutrients from wastewater in relation with medium carbon and nitrogen sources were described in Table 3b. The variation demonstrated the difference of bacteria in nitrification and denitrification. Yang et al. (2011) reported denitrifying and nitrifying property of *Bacillus subtilis* A1. This organism has been showed COD removal efficiencies of 63.9 ± 1.8%, 64.5 ± 1.5%, 67.1 ± 0%, and 71.0 ± 0.5%, respectively in the culture medium containing succinate, citrate, glucose, and acetate, respectively. Chen and Ni (2012) reported ammonium removal efficacy of *Agrobacterium* sp. LAD9 by the process of aerobic denitrification - heterotrophic nitrification. Heterotrophic microorganisms easily assimilated small and simple sugars and organic acids than highly complex molecules (Chen and Ni, 2012).

**Table 3a:** Removal of nutrients by *P. aeruginosa* NU1 from the wastewater at various concentrations of carbon and nitrogen sources.

Independent	Variables	% Removal			
Factors		NH_4_^+^ - N	NO_3_^−^ - N	NO_2_^−^ - N	PO_4_^3−^ = _
Glucose (%)	1	89.3 ± 3.2	78.3 ± 1.6	65.5 ± 1.3	82.4 ± 2.6
	2	92.3 ± 1.09	86.3 ± 2.2	78.5 ± 2.9	85.4 ± 3.3
	3	94.2 ± 0.8	87.3 ± 3.7	81.3 ± 3.2	88.6 ± 2.8
	4	90.3 ± 1.1	90.9 ± 2.2	82.8 ± 1.9	90.4 ± 2.2
	5	89.4 ± 1.9	84.3 ± 1.9	80.5 ± 1.7	79.5 ± 1.7
Ammonium Nitrate (%)	0.1	90.4 ± 3.8	87.6 ± 2.3	93.2 ± 2.2	86.3 ± 4.5
	0.2	87.5 ± 1.1	85.3 ± 2.2	91.4 ± 5.4	90.3 ± 3.3
	0.3	85.3 ± 1.7	84.1 ± 1.1	79.5 ± 1.7	91.5 ± 4.2
	0.4	79.5 ± 3.6	80.5 ± 2.4	76.3 ± 4.8	86.3 ± 1.2
	0.5	75.6 ± 1.8	76.1 ± 5.3	70.4 ± 1.6	83.2 ± 0.9
C:N ratio	1:1	89.4 ± 1.4	89.4 ± 2.7	90.3 ± 2.3	85.4 ± 5.8
	2:1	95.2 ± 2.9	93.5 ± 2.6	92.1 ± 4.7	84.1 ± 1.1
	1:2	90.3 ± 0.9	85.3 ± 1.1	87.5 ± 3.9	75.4 ± 0.9
	2:2	85.5 ± 1.3	84.1 ± 2.5	84.3 ± 4.5	74.3 ± 1.3

**Table 3b:** Removal of nutrients by A. calcoaceticus K12 from the wastewater at various concentrations of carbon and nitrogen sources.

Independent	Variables	% Removal			
Factors		NH_4_^+^ - N	NO_3_^−^ - N	NO_2_^−^ - N	PO_4_^3−^ = _
Glucose (%)	1	76.5 ± 4.4	65.6 ± 4.7	79.5 ± 5.2	78.5 ± 4.8
	2	80.5 ± 5.5	70.7 ± 5.1	84.5 ± 5.9	87.4 ± 2.9
	3	78.5 ± 4.7	67.9 ± 8.5	81.3 ± 4.1	86.4 ± 1.4
	4	67.5 ± 0.65	65.3 ± 1.9	75.1 ± 0.62	76.2 ± 3.8
	5	65.4 ± 4.7	60.7 ± 0.5	73.2 ± 1.4	70.4 ± 6.8
Ammonium Nitrate (%)	0.1	89.5 ± 5.9	57.8 ± 0.86	69.6 ± 1.5	67.5 ± 2.9
	0.2	90.3 ± 6.3	70.9 ± 7.4	75.2 ± 3.2	76.1 ± 1.5
	0.3	87.5 ± 1.6	69.7 ± 2.7	70.3 ± 2.2	75.3 ± 2.4.
	0.4	85.3 ± 0.52	67.3 ± 4.5	65.3 ± 3.8	70.5 ± 2.5
	0.5	82.1 ± 0.47	54.4 ± 2.8	58.5 ± 5.7	65.8 ± 2.9
C:N ratio	1:1	68.7 ± 3.4	53.7 ± 1.3	43.5 ± 4.4	78.3 ± 2.5
	2:1	76.5 ± 0.8	87.6 ± 2.9	74.2 ± 2.2	81.4 ± 2.7
	1:2	82.1 ± 0.58	89.5 ± 3.3	84.2 ± 5.8	84.5 ± 4.2

### 3.5 Effect of temperature and pH on nutrient removal by bacterial strains

Temperature variation showed significant effect on nutrient removal property. With the raise of incubation temperature, the nutrient removal efficiency improved up to 40 °C and further increase of temperature gradually decreased nutrients removal due to the suppression of enzymatic activities (Table 4a). At higher pH values nutrients removal efficacy was increased two-fold (Table 4b). The bacteria such as *Enterobacter* sp. FL (Wang et al., 2018), *Acinetobacter* sp. SZ28 (Suet al., 2015), and *Pseudomonas stutzeri* strain T1 (Guo et al., 2013) have been used to remove nutrients from the wastewater and varying nutrient removal ability was reported.

**Table 4a:** Removal of nutrients by P. aeruginosa NU1 from the wastewater at various concentrations of carbon and nitrogen sources.

Independent	Variables	% removal			
Factors		NH_4_^+^ - N	NO_3_^−^ - N	NO_2_^−^ - N	PO_4_^3−^ = _
Temperature (°C)	25	68.4 ± 0.71	78.5 ± 7.1	75.2 ± 1.9	69.4 ± 3.9
	30	70.3 ± 1.6	80.4 ± 2.9	86.3 ± 2.7	72.5 ± 1.4
	35	74.2 ± 0.52	85.2 ± 0.41	89.9 ± 6.2	76.9 ± 2.1
	40	87.4 ± 0.26	89.4 ± 1.7	91.4 ± 3.2	85.7 ± 1.1
	45	84.2 ± 5.2	85.9 ± 3.1	85.6 ± 4.9	82.4 ± 0.28
pH	6	43.4 ± 1.8	67.4 ± 1.9	67.4 ± 3.3	58.5 ± 1.6
	6.5	51.9 ± 3.3	79.3 ± 4.3	70.4 ± 5.8	65.3 ± 8.4
	7	54.2 ± 2.6	80.2 ± 5.7	78.5 ± 2.8	74.4 ± 3.9
	7.5	60.3 ± 1.8	84.3 ± 5.8	80.2 ± 3.7	76.1 ± 3.8
	8	88.4 ± 0.43	85.4 ± 3.9	81.3 ± 1.9	80.5 ± 1.1
	8.5	83.2 ± 1.5	19.6 ± 1.5	75.9 ± 4.3	78.4 ± 3.1

**Table 4b:** Removal of nutrients by A. calcoaceticus K12 from the wastewater at various concentrations of carbon and nitrogen sources.

Independent	Variables	% Removal			
Factors		NH_4_^+^ - N	NO_3_^−^ - N	NO_2_^−^ - N	PO_4_^3−^ = _
Temperature (°C)	25-	69.6 ± 0.6	83.2 ± 4.8	47.3 ± 1.3	63.2 ± 1.6
	30-	75.4 ± 0.67	87.3 ± 0.62	51.3 ± 2.9	74.9 ± 2.8
	35-	80.5 ± 0.27	91.4 ± 0.52	63.2 ± 2.6	82.7 ± 3.3
	40-	86.2 ± 0.56	95.4 ± 1.63	83.2 ± 1.8	85.8 ± 2.1
	45-	80.1 ± 0.37	89.5 ± 2.62	78.2 ± 2.4	79.3 ± 4.5
pH	6	50.5 ± 0.59	64.9 ± 1.9	47.3 ± 3.3	68.4 ± 2.6
	6.5	52.1 ± 0.79	74.8 ± 1.4	49.2 ± 2.8	70.5 ± 3.2
	7	59.5 ± 4.7	89.3 ± 3.2	78.4 ± 2.6	75.3 ± 8.1
	7.5	89.6 ± 1.6	92.4 ± 2.9	87.4 ± 2.9	85.1 ± 3.8
	8	86.5 ± 1.3	90.01 ± 1.5	78.5 ± 6.2	84.7 ± 1.9
	8.5	84.2 ± 0.86	76.5 ± 0.4	69.4 ± 2.2	76.3 ± 4.3

### 3.6 Wastewater treatment in moving bed biofilm reactor

**Fig. 1:**
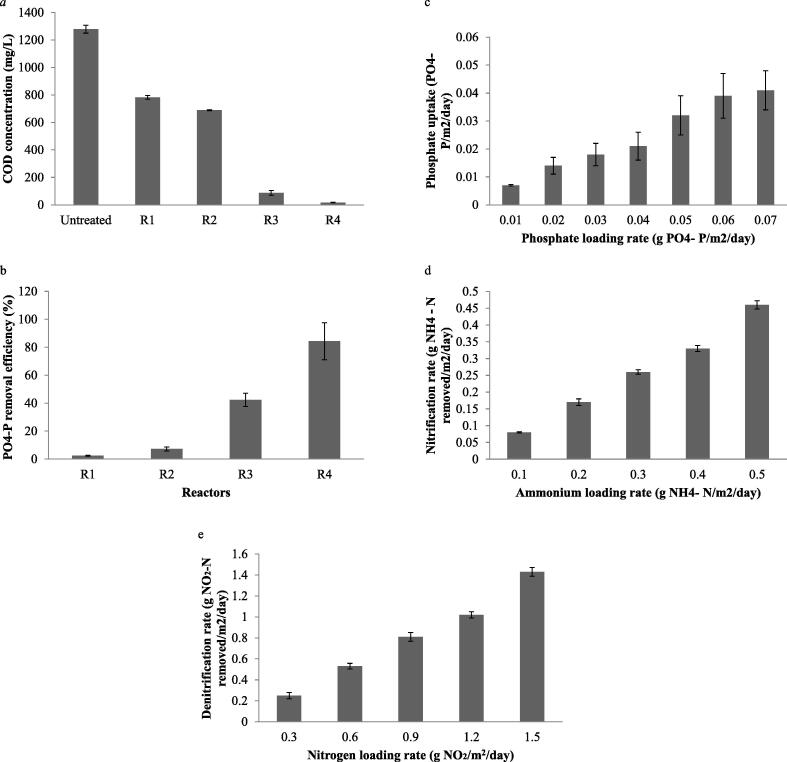
Removal of nutrients in the MBBRs. (a) COD level of wastewater during treatment in various stages of MBBRs, (b) removal of phosphate from the wastewater in various reactions in MBBR, (c) phosphate uptake in the aerobic bioreactor, (d) nitrification rate of nutrients in the reactor at various ammonia concentrations, (e) Denitrification rate in the reactor at various concentrations of nitrogen.

The characteristics of wastewater applied in MBBR were pre-determined and the results were described in supplementary Table 1 (Table S1). Municipal sewage treatment plants receive a multitude of contaminants present from various sources, hospitals to industries. Both organisms have the ability to withstand without any dilution of wastewater. In this study, the selected bacterial strains were inoculated and biofilms were allowed to mature in biocarriers for 12 days. The bacterial strains adhered on carriers filled in the aerobic reactor. Bacteria have the ability to form biofilm at varying level based on the biocarriers. Bacterial biofilms formed on MBBR biocarriers could improve the functional properties of bacteria, allowing simultaneous removal of nitrogen and COD simultaneously and synergistic properties have also been reported previously (Ma et al., 2018). The variation of COD profile of wastewater at four different reactors of MBBR laboratory model is described in Fig. 1a. According to the findings, the results revealed 98.7% organic carbon removal in MBBR system. As seen from the results, the mean COD level in R1, R2, R3 and R4 reactors were, 782 ± 13.6 mg/L, 698 ± 3.2 mg/L, 87.3 ± 17.8 mg/L, 16.8 ± 2.2 mg/L, respectively. Thus, in the R4 reactor (aerobic reactor) COD load was significantly lower than R1 (61.1%), R2 (6.83%), R3 (54%) and R4 (1.9%), respectively. Phosphorus removal efficiency and phosphorus accumulating organisms play significant role in phosphate removal in the reactors. In anaerobic reactors biological phosphorus removal property is initiated and phosphorus accumulating bacteria converted acetate into carbon. The present findings indicated that the designed MBBR reactors has acceptable level phosphorus removal properties up to 7.2 ± 3.8%, 42.4 ± 4.6%, and 84.2 ± 13.1% in the reactors R1, R2, R3 and R4, respectively (Fig. 1b). According to Fig. 1c, phosphate uptake was 0.009 PO^4^-P/m^2^/day at 0.01 PO^4^-P/m^2^/day loading rate and uptake was maximum (0.041 PO^4^-P/m^2^/day) at 0.07 PO^4^-P/m^2^/day loading rate. In the anaerobic reactor, phosphorus accumulating organisms utilized volatile fatty acids from the wastewater and reduced COD level. Hence, the level of acetate in the anaerobic reactor influenced microbial cell growth and improved phosphorus removal. The biofilm producing organisms (*P. aeruginosa NU1* and A. calcoaceticus K12) used in this study involved in nitrification process. In aerobic bioreactor (R4), nitrification rate was positively correlated to ammonia loading rate in the reactor. At 0.1 g NH_4-_ -N/m^2^/day loading rate, nitrification rate was 0.08 NH_4-_ -N/m^2^/day and improved as 0.46 NH_4-_ -N/m^2^/day at 0.5 NH_4-_ -N/m^2^/day loading rate (Fig. 1d). The rate of denitrification in relation to nitrogen loading rate in the reactor was described in Fig. 1e. Denitrification rate showed linear relationship at increasing concentrations nitrogen content in the reactor and denitrification rate was 1.43 g NO_2_-N /m^2^/day at 1.5 g NO_2_-N /m^2^/day. COD removal rate was achieved over 90% during the treatment at high salt concentration in the bioreactor. At higher organic load in the wastewater decreased nitrate level indicated collapse in the nitrification process. The removal efficacy of total nitrogen was higher than previous findings. Lin (2018) observed 72% total nitrogen removal rate in sewage system and only 58% total nitrogen removal was reported by Zubrowska-Sudol and Walczak (2015).

### 3.7 Dehydrogenase activity

**Fig. 2:**
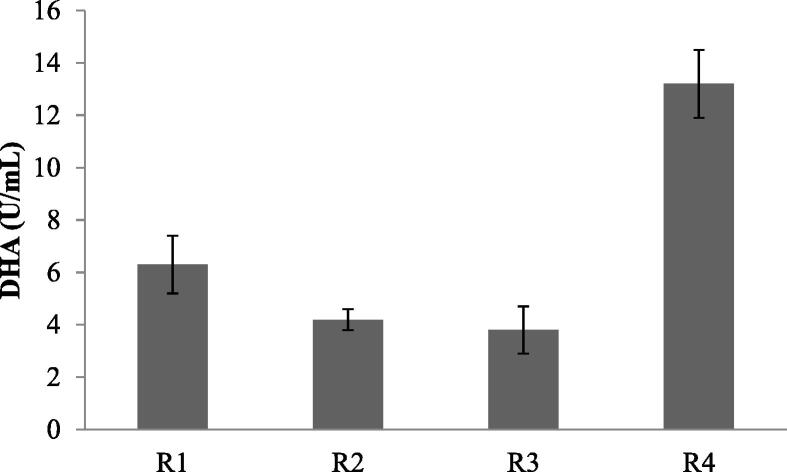
DHA activity in various reactors in MBBR. R1-anaerobic reactor, R2 and R3 – anoxic reactor, R4 – aerobic reactor.

The amount of DHA in the sample was evaluated from the bioreactor and described in Fig. 2. DHA activity was high in aerobic reactor R4 than R1, R2 and R3 reactors. The high DHA activity indicated good metabolic activity by microbes in R4 reactor. However, the metabolic activity can be affected by various biological and physical factors. Bacteria in the bioreactor are the important source in the biological process, and oxidation of organic compounds takes place mainly due to the enzyme activities (Vijayaraghavan et al., 2016). Hence, measuring enzyme activities in the bioreactors could be a potential alternative for analyzing the performance of microorganisms. In the bioreactor, maintaining optimal concentration of DHA could help the bioreactor for better degradation of organic substances (Pourakbar et al., 2020).

## 4 Conclusions

Two indigenous bacterial strains, *Pseudomonas aeruginosa* NU1 and Acinetobacter calcoaceticus K12 were isolated from the municipal soil sludge. These strains have the ability to synthesize0.87 ± 0.05 U/mg and 0.52 ± 0.12 U/mg hydroxylamine oxidase, 1.023 ± 0.062 U/mg and 1.29 ± 0.07 U/mg nitrite reductase, and 0.789 ± 0.031 U/mg and 1.07 ± 0.13 U/mg nitrate reductase, respectively. These strains can tolerate various wastewater shocks, including, antibiotic and utilized carbon sources for biomass production. Biomass production was optimum and was not affected without any pre-treatment. Anaerobic and anoxic condition with biofilm developed by *P. aeruginosa* NU1 and A. calcoaceticus K12 in the bioreactor removed 98.7% organic matters from the wastewater. COD removal efficiency was improved in the reactor after degradation of organic wastes. Available phosphate in the wastewater was utilized by these microorganisms using various metabolic pathways based on the availability of nutrients. Based on the present findings, MBBRs are useful for the degradation domestic wastewater with minimum working area.

## Declaration of Competing Interest

The authors declare that they have no known competing financial interests or personal relationships that could have appeared to influence the work reported in this paper.
