# Supplementary material for: Biofilm producing indigenous bacteria isolated from municipal sludge and their nutrient removal ability in moving bed biofilm reactor from the wastewater
Source: Saudi J Biol Sci. 2021 Jul 1;28(9):4994–5001. doi: 10.1016/j.sjbs.2021.06.084 (PMC8381082; doi:10.1016/j.sjbs.2021.06.084)
Supplement: Supplementary data 1 [file mmc1.docx]

# Table S1. Physico-chemical properties of wastewater from inlet and outlet of the reactor. Physico-chemical properties of water in inlet and treated wastewater collected from the outlet.

| Parameters and units | Inflow | Outflow |
| --- | --- | --- |
| pH | 8.42 ± 0.2 | 7.32 ±0.1 |
| BOD (mg/mL) | 957 ±29.3 | 242 ± 2.7 |
| COD (mg/L) | 1272 ± 28.4 | 482 ± 10.3 |
| TSS (mg/L) | 1630 ± 20.2 | 539 ± 39.4 |
| Al (mg/L) | 0.02 ± 0.0 | ND |
| Fe (mg/L) | 0.82 ± 0.01 | ND |
| Cd (mg/L) | 0.001 ± 0.0 | ND |
| Total phosphorus (mg/L) | 1.8 ± 0.52 | 0.2 ± 0 |
| Nitrate (mg/L) | 2.1 ± 0.65 | 0.12 ± 0.02 |
| Nitrite (mg/L | 0.65 ± 0.21 | 0.11 ± 0.03 |
| P-PO_4_ | 2.8 ± 0.18 | 1.3 ± 0.06 |

# NA-None detected
